# Psychometric characteristics of the Functional Assessment of Cancer Therapy-General when applied to Brazilian cancer patients: a cross-cultural adaptation and validation

**DOI:** 10.1186/s12955-015-0400-8

**Published:** 2016-01-13

**Authors:** Juliana Alvares Duarte Bonini Campos, Maria Cláudia Bernardes Spexoto, Sergio Vicente Serrano, João Maroco

**Affiliations:** Departamento de Alimentos e Nutrição. Faculdade de Ciências Farmacêuticas de Araraquara, UNESP-Univ Estadual Paulista, Rod. Araraquara-Jaú, km 01, Araraquara, São Paulo Brazil; Barretos Cancer Hospital - Fundação Pio XII, Barretos, São Paulo Brazil; William James Center for Research, ISPA-Instituto Universitário, Rua Jardim do Tabaco, n°34, 1149-041 Lisbon, Portugal

**Keywords:** Cancer, Validity, Reliability, Brazil, Health-related quality of life

## Abstract

**Background:**

The psychometric properties of an instrument should be evaluated routinely when using different samples. This study evaluated the psychometric properties of the Functional Assessment of Cancer Therapy-General (FACT-G) when applied to a sample of Brazilian cancer patients.

**Methods:**

The face, content, and construct (factorial, convergent, and discriminant) validities of the FACT-G were estimated. Confirmatory factor analysis (CFA) was conducted the ratio chi-square by degrees of freedom (*χ*^2^/df), the comparative fit index (CFI), the Tucker-Lewis index (TLI), and the root mean square error of approximation (RMSEA) as indices. The invariance of the best model was assessed with multi-group analysis using the difference of chi-squares method (Δχ^2^). Convergent validity was assessed using Average Variance Extracted (AVE) and discriminant validity was determined via correlational analysis. Internal consistency was assessed using the Cronbach’s alpha (α) coefficient, and the Composite Reliability (CR) was estimated.

**Results:**

A total of 975 cancer patients participated in the study, with a mean age of 53.3 (SD = 13.0) years. Of these participants, 61.5 % were women. In CFA, five correlations between errors were included to fit the FACT-G to the sample (*χ*^2^/df = 8.611, CFI = .913, TLI = .902, RMSEA = .088). The model did not indicate invariant independent samples (Δχ^2^: μ: p < .001, i: p < .958, Cov: p < .001, Res: p < .001). While there was adequate convergent validity for the physical well-being (AVE = .54) and social and family Well-being factors (AVE = .55), there was low convergent validity for the other factors. Reliability was adequate (CR = .76–.89 and α = .71–.82). Functional well-being, emotional well-being, and physical well-being were the factors that demonstrated a strong contribution to patients’ health-related quality of life (β = −.99, .88, and .64, respectively).

**Conclusion:**

The FACT-G was found to be a valid and reliable assessment of health-related quality of life in a Brazilian sample of patients with cancer.

## Background

Health-related quality of life (HRQOL) is a concept that has assumed an important role in clinical practice [1–7] and research aimed at cancer patients [3, 8, 9]. The disorganization of the HRQOL of patients with cancer can have a significant negative impact on treatment response, symptoms associated with the disease/treatment, disease prognosis, and the development of unpleasant concomitant clinical conditions. The improvement of procedures and diagnostic techniques as well as the variety of available treatment modalities have led to increased life expectancy in cancer patients, leading to greater concern about their long-term well-being [10]. Thus, when choosing between available therapies, the oncologist’s decision-making is driven not only by the effectiveness of the treatment, but also by its impact on HRQOL [11].

However, since HRQOL is a latent variable, (i.e., not directly measurable), many instruments have been developed to evaluate it in the general population (e.g., World Health Organization Quality Of Life Assessment [WHOQOL-100] [12–16], Medical Outcomes Study SF - 36-Short Form Health Survey [17, 18], and the Sickness Impact Profile [19]).

Given the increase in generic measures for assessing HRQOL, there is a need for instruments that are specific to individuals’ clinical conditions and that consider associated disabilities, limitations, and symptoms specific to each condition. Among the instruments proposed in the literature to assess HRQOL in cancer patients, the two most frequently used instruments used worldwide are the Cella et al.’s Functional Assessment of Cancer Therapy-General (FACT-G) [10] and the European Organization for Research and Treatment of Cancer-Quality of Life Questionnaire Core 30 (EORTC QLQ-C30) [20].

Initially published in 1993 in the United States, the FACT-G, first has been translated into more than 30 languages [21]. To determine its suitability for use in different countries and samples, a number of validation studies have been conducted [10, 22, 23].

Despite the existence and widespread application of a Portuguese version of the FACT-G, only one study [24] has evaluated the psychometric properties of this instrument. However, this study was limited to evaluation of the FACT-G psychometric properties in a sample of individuals with cervical cancer.

Moreover, it is important to note that despite the fact that existing literature [10, 25] indicates that the FACT-G is appropriate for use in oncology, easy to use, concise, and responsive to clinical changes, its psychometric properties should be routinely evaluated when applied to different samples. Specifically, this should occur because of knowledge that these properties are unrelated to the instrument itself, but rather to the sample, and is thereby directly influenced by the sample’s characteristics [26].

Thus, we performed this study to evaluate the psychometric properties of the FACT-G when applied to a large sample of Brazilian cancer patients.

## Methods

### Study design and sampling

The present study employed a cross-sectional design. A total of 1099 cancer patients from the outpatient clinic and in the hospitalization sector of the Barretos Cancer Hospital were invited to participate in this study during 2013.

The estimated minimum sample size was based on Hair et al.’s [27] recommendation of 5–10 subjects (k) for each model parameter. Since the tested model has 58 parameters (FACT-G: 27 items, 27 errors, and 4 factors), we estimated that 290–580 subjects would be required. As the study also evaluated the invariance of the instrument for independent samples, it was necessary to use a second sample of the same size. Thus, the minimum sample size needed to conduct the required analysis was between 580 and 1160 participants. However, since another objective was to explore the psychometric properties of the FACT-G for the population of cancer patients in Brazil, the sample had to be large enough to conveniently capture the population’s variability. Consequently, we chose to analyze data from a representative sample of 1020 participants.

The exclusion criteria for participants were as follows: had undergone a surgical procedure, had been diagnosed with cognitive impairment, were undergoing palliative care, had been diagnosed with a severe psychiatric disorder, and under 18 years of age. We included all adult patients who agreed to participate, regardless of cancer diagnosis, treatment type and clinical stage, as well as having no previously reported exclusion characteristics.

### Study variables and measures

To characterize the sample, we collected socio-demographic and clinical information (Table 1). It is important to clarify that the socio-economic class and household head education level were classified according to the Associação Brasileira de Empresas de Pesquisa (ABEP) [28], and that clinical information about the disease was obtained by consulting the patient’s chart.

**Table 1:** Socio-demographic and clinical characterization of participants^a^

Socio-demographic characteristics	n	%	Clinical characteristic	n	%
Gender			Specialty		
Male	375	38.5	Head and neck	73	7.5
Female	600	61.5	Higher digestive tract	94	9.6
Religion			Lower digestive tract	200	20.5
No	40	4.2	Gynecology	107	11.0
Yes	922	95.8	Hematology	4	.4
Religious Practice			Breast Cancer	297	30.5
No	139	14.5	Brain tumor	14	1.4
Yes	822	85.5	Orthopedic	24	2.5
Marital status			Skin	41	4.2
Single	139	14.3	Thorax	48	4.9
Married	631	64.8	Urology	73	7.5
Widowed	99	10.2	Clinical stage		
Separated/Divorced	104	10.7	I	72	8.4
Working			II	215	25.1
No	716	73.6	III	333	38.8
Yes	257	26.4	IV	237	27.7
Economic class			Treatment type		
A	24	2.4	Chemotherapy	616	63.4
B	334	34.3	Radiotherapy	163	16.8
C	462	47.4	Chemotherapy and radiotherapy	148	15.2
D and E	155	15.9	Hormone therapy	26	2.7
Data collection place			Immunotherapy	18	1.9
Outpatient	29	3.0	Metastasis		
Hospitalization units	940	97.0	No	599	61.8
			Yes	370	38.2
			Body Mass Index (kg/m^2^)		
			<18.5 (Low weight)	57	5.9
			18.5├25.0 (Eutrophic)	407	42.3
			25.0├30.0 (Pre obesity)	310	32.2
			≥30.0 (Obesity)	188	19.5

^a^%: were calculated for each variable, considering the total number of responders and non-participants

The height (cm) and weight (kg) reported by patients were recorded for the calculation of body mass index (kg/m^2^) (BMI) and the subsequent classification of their nutritional status. For BMI classification, we used the adult cutoff points (>20 years) proposed by the World Health Organization [29].

The average age of the 1020 participants was 53.3 (SD = 13.0) years, and all had an established cancer diagnosis.

It is important to highlight that not all patients answered all questions on the socio-demographic inventory, and that some patients’ charts did not contain some clinical information. Additionally, it should be clarified that the lowest response rate was for information about clinical stage (88.0 %), which was related to the clinical difficulty of establishing such precise stage-related information rather than a lack of information in the medical record. However, since response rates for socio-demographic variables (98.5–100.0 %) and clinics (98.6–100 %) were high (with the exception of clinical stage), this does not represent a study limitation.

HRQOL was estimated using the FACT-G [10]. The FACT-G was first published in 1993 in the United States and was developed based on structured interviews with oncology experts and cancer patients. The use of instrument was proposed following five years of development and psychometric assessments [10]. Factor analysis of the instrument, originally developed to consist of 28 items, revealed the following five factors [10].

In 1999, Webster et al. [21] conducted a study in mixed sample of cancer patients (*n* = 99), and proposed the removal of one factor (“Relationship with the doctor”) and its associated items. This decision was made after evaluation of cultural adaptations of the instrument, patient feedback, and statistical analysis using item response theory (IRT). However, one additional item was added (“I am concerned that my condition will get worse”). Based on these findings and proposals, the current FACT-G is now in its fourth version.

Thus, in this study, we used the fourth version of the instrument proposed by Webster et al. [21], which is comprised of 27 items divided into four factors (physical well-being, social and family well-being, emotional well-being, functional well-being) in a reflexive model. All items are rated using a 5-point Likert scale response format (0 = Not at all, 4 = Very much). Two factors are formulated in the positive direction and two in the negative direction. One item in the emotional well-being dimension presents a reversed response scale relative to the other items for the same factor. In this study, we used the Portuguese version of the FACT-G, which was provided and authorized by the Functional Assessment of Chronic Illness Therapy (FACIT) Group.

The non-response rate to FACT-G items (missing data) ranged from 0.1 to 0.6 % with the exception of item 14, which had an extremely high non-response rate (45 %). Considering this item refers to “satisfaction with sex life” and the literature has reported that participants infrequently respond to this item [30] and that FACIT permits its exclusion, we decided to remove this item from the analysis. Additionally, since the sample had an expanded size, it was decided to exclude individuals who did not respond to one or more of the other instrument items (listwise). Thus, the final sample consisted of 975 participants (mean age: 53.3 years, SD = 13.0) (Table 1).

In this study, we also used the EORTC QLQ-C30 to estimate the concurrent validity of the FACT-G. Therefore, it should be clarified that used the original factorial proposal for the EORTC QLQ-C30 consists of nine first order factors (Physical, Role, Emotional, Cognitive and Social functioning, Fatigue, Nausea and vomiting, Pain, Overall Quality of Life - global score) and 5 single terms grouped together into a factor referred to as “Spurious.” The fit of this version to sample was adequate (*χ*^2^/df = 3071; Comparative Fit Index [CFI] = .968; Tucker-Lewis index [TLI] = .961; root mean square error of approximation [RMSEA] = .046).

### Transcultural adaptation of the FACT-G

#### Face validity

To analyze face validity, we used the guidelines proposed by Guillemin et al. [31] and Beaton et al. [32].

The instrument translation was independently performed by three bilingual translators who were native Portuguese speakers and had knowledge and experience in an English-speaking country. This study’s researchers evaluated translations in order to obtain a consensus for a single Portuguese version. This version was back-translated by a bilingual individual who was a native English speaker. The final Portuguese version was then evaluated by a team of three teachers and three Portuguese oncology experts to ensure that the semantic, idiomatic, cultural, and conceptual aspects of the instrument were comparable to those of the original version.

Our final version was compared to the original version of the instrument and to the original Portuguese version proposed by the FACIT Group. It was not necessary to alter the Portuguese version presented by FACIT.

To calculate the misunderstanding index (MI) of each item, the FACT-G was administered to the same 32 cancer patients in interview form. No items presented a MI > 10 %; thus, it did not require reformulation.

#### Content validity

The content validity of the FACT-G was estimated using the Content Validity Ratio (CVR) proposed by Lawshe [33]. In this stage, 12 judges/experts in the field of oncology/nutrition rated each item according to its essentiality. To evaluate the CVR, we used Wilson et al.’s [34] proposal of α = 5 % (CVR_12; 0.05_ ≥ 0.57).

### Evaluation of psychometric characteristics

To evaluate the psychometric sensitivity of the items, summary and shape measures were estimated. Items with absolute values of skewness (Sk) and kurtosis (Ku) greater than 3 and 7, respectively [26], were considered to present a severe violation of normality. Multivariate outliers were identified using Mahalanobis distance [35, 36].

The construct validity of the instrument was estimated by measuring factorial, convergent, and discriminant validity. We conducted confirmatory factor analysis (CFA) using the matrix of polychoric correlations and the Weighed Least Squares Mean and Variance Adjusted (WLSMV) using MPLUS 6.12 software (Muthén & Muthén, Los Angeles, CA). To evaluate the goodness of fit, we used as the ratio chi-square by degrees of freedom (*χ*^2^/df), CFI, TLI, and RMSEA as indices [35]. The model’s fit was considered adequate when *χ*^2^/df ≤ 2.0, CFI and TLI ≥ .9, and RMSEA ≤ .08 [27, 35].

Items that had factor weights (λ) < .40 were removed, as well as those that were redundant by the modification indices estimated by the Lagrange multipliers (LM) method (LM > 11, p < .001). The modification indices were also used to verify the correlation between the item errors [35].

The invariance of the best model was assessed with multi-group analysis using the difference of chi-squares method (Δχ^2^). Here, the sample was randomly divided into two parts (6:4), where 60 % comprised the “Test Sample” and 40 % the “Sample Validation.” Multi-group analysis used to conduct the invariance test, using the chi-square difference (Δχ^2^) between the model with free factorial weights and the model with equal weights. If the hypothesis of the factorial weight’s invariance was acceptable (metric invariance), then analyses of the intercepts’ invariance (scalar invariance) and residual’s variances/covariances invariance (structural invariance) were conducted [26, 37].

Correlation analysis between the factors of the FACT-G and EORTC QLQ-C30 were used to estimate the concurrent/divergent validity. The concurrent validity is determined by a front moderate to strong correlation between theoretically similar factors and divergent validity by a front of weak correlations among theoretically distinct factors.

Convergent validity was assessed using the Average Variance Extracted (AVE) [35, 38] and values of AVE ≥ .50 were considered adequate [27]. Discriminant validity was estimated by calculating the correlations between the factors and was considered adequate when AVEi and AVEj ≥ ρ_ij_^2^ [26, 35, 38].

We assessed internal consistency using Cronbach’s standardized alpha coefficient (α) and Composite Reliability (CR). Internal consistency was considered adequate when α ≥ .70 [39] and CR ≥ .70 [27].

After defining the best model, we explored the presence of a significant correlation between the factors. Subsequently, based on the theoretical justification for evaluation of a higher order factor, we developed a second order hierarchical model (SOHM).

The analyses were performed using SPSS version 22.0 (IMB, Chicago, IL) and MPLUS 6.12 (Muthén & Muthén, Los Angeles, CA) programs.

### Ethical considerations

The present study was approved by the Research in Humans Ethics Committee of the Cancer Hospital of Barretos (protocol 561/2011). Only patients who agreed and signed the informed consent form participated in the study.

## Results

The adherence rate was 92.8 %. The reasons for non-adherence to the study were lack of time (*n* = 5), having participated in another study on the same day (*n* = 1), shyness (*n* = 2), not feeling well (*n* = 3), refusal to complete the socio-demographic questionnaire (*n* = 1), no justification (*n* = 7), and reluctance to participate because data were to be collected via interview (*n* = 60).

Among participants, there was a higher prevalence of women as well as individuals with religious practice and those who were married, working, had breast and lower digestive tract cancer, were undergoing chemotherapy, and without metastasis (Table 1).

The summary measures of participants’ answers on the FACT-G and the CVR are presented in Table 2.

**Table 2:** Summary and shape measures and the content validity ratio of the items

FACT-G	Mean	Median	Mode	Standard-deviation	Skewness	Kurtosis	CVR^a^
FACT1	.83	0	0	1.14	1.19	.34	.50
FACT2	.50	0	0	.96	2.04	3.37	.17
FACT3	.50	0	0	1.01	2.02	3.04	-.33
FACT4	.90	0	0	1.14	1.02	-.04	1.00^b^
FACT5	.66	0	0	1.14	1.63	1.41	.67^b^
FACT6	.43	0	0	.86	2.03	3.39	.33
FACT7	.56	0	0	1.08	1.79	1.92	.00
FACT8	2.98	3	3	1.06	−1.17	.95	.00
FACT9	3.49	4	4	.72	−1.69	3.81	.50
FACT10	3.12	3	4	.99	−1.30	1.49	.50
FACT11	3.19	3	3	.80	−1.59	4.23	−.33
FACT12	3.43	4	4	.73	−1.94	6.19	−.33
FACT13	3.59	4	4	.57	−1.64	5.68	.33
FACT14	2.46	3	3	1.28	−.68	−.62	.33
FACT15	.79	0	0	1.13	1.28	.51	.67^b^
FACT16	.71	1	1	.67	1.32	4.66	.33
FACT17	.20	0	0	.65	3.63	13.14	.83^b^
FACT18	.60	0	0	1.05	1.75	2.02	.17
FACT19	.30	0	0	.85	3.02	8.36	.33
FACT20	.56	0	0	1.06	1.89	2.45	.50
FACT21	2.39	3	3	1.20	−.54	−.59	.33
FACT22	2.73	3	3	1.12	−1.17	.79	−.17
FACT23	3.02	3	3	.91	−1.11	1.47	.17
FACT24	3.08	3	3	.78	−1.77	5.40	.67^b^
FACT25	2.62	3	3	1.15	−.96	.21	.50
FACT26	3.05	3	3	.75	−1.54	4.76	.17
FACT27	3.01	3	3	,83	−1.17	2.50	.83^b^

^a^ CVR_12; 0.05_ = 0.57; ^b^ values above the significant value (essential items)

The only item that presented psychometric sensitivity issues was item 17 (“losing hope in the fight”). We noticed that the judges/experts indicated that only 6 items were considered essential to assessment of the impact of disease/treatment on the HRQOL of cancer patients.

The factor structure of the FACT-G showed unsatisfactory fit to the sample (λ = .43 to .92; *χ*^2^/df = 13.313, CFI = .857, TLI = .841, RMSEA = .112). Following the modification indices, five correlations were inserted between the errors (e8–e10, e11–e12, e12–e13, e19–e20, e21–e22).

**Fig. 1:**
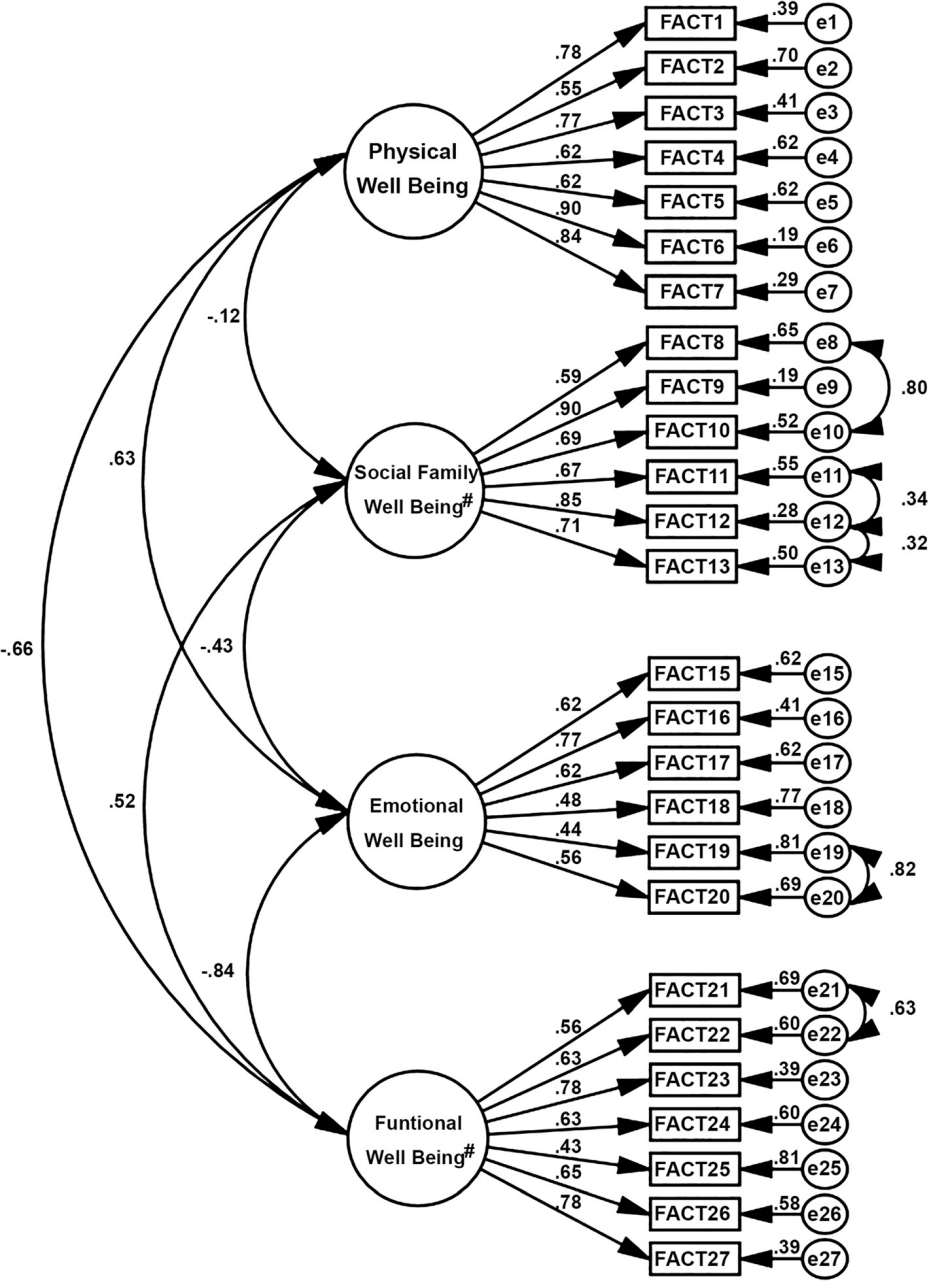
Factor structure of the refined model of the Functional Assessment of Cancer Therapy. λ = .43 to .90; *χ* ^2^/df = 8.611, Comparative fit index (CFI) = .913, Tucker-Lewis index (TLI) = .902, Root mean square error of approximation (RMSEA) = .088 fitted to the sample of Brazilian cancer patients

The factor structure of the refined model fit the sample (Appendix) (λ = .43 to .90; *χ*^2^/df = 8.611, CFI = .913, TLI = .902, RMSEA = .088) and is presented in Fig. 1.

The fit of the refined model was adequate for the test sample (λ = .42 to .89; *χ*^2^/df = 5.034, CFI = .914, TLI = .903, RMSEA = .083) and for the validation sample (λ = .40 to .89; *χ*^2^/df = 4.436, CFI = .916, TLI = .905, RMSEA = .094). The model was not invariant for independent samples (Δχ^2^: μ: *p* < .001, i: *p* < .958, Cov: *p* < .001, Res: *p* < .001).

We observed low convergent validity for emotional well-being (AVE = .35), and functional well-being (AVE = .42). The AVE presented adequate values for physical well-being (AVE = .54) and social and family well-being factors (AVE = .55). The reliability was adequate (CR = .76–.89 and α = .71–.82).

Based on the significant correlations between the primary factors (Fig. 1) and the theoretical justification of drafting a final construct built from the four primary factors, a SOHM was constructed.

**Fig. 2:**
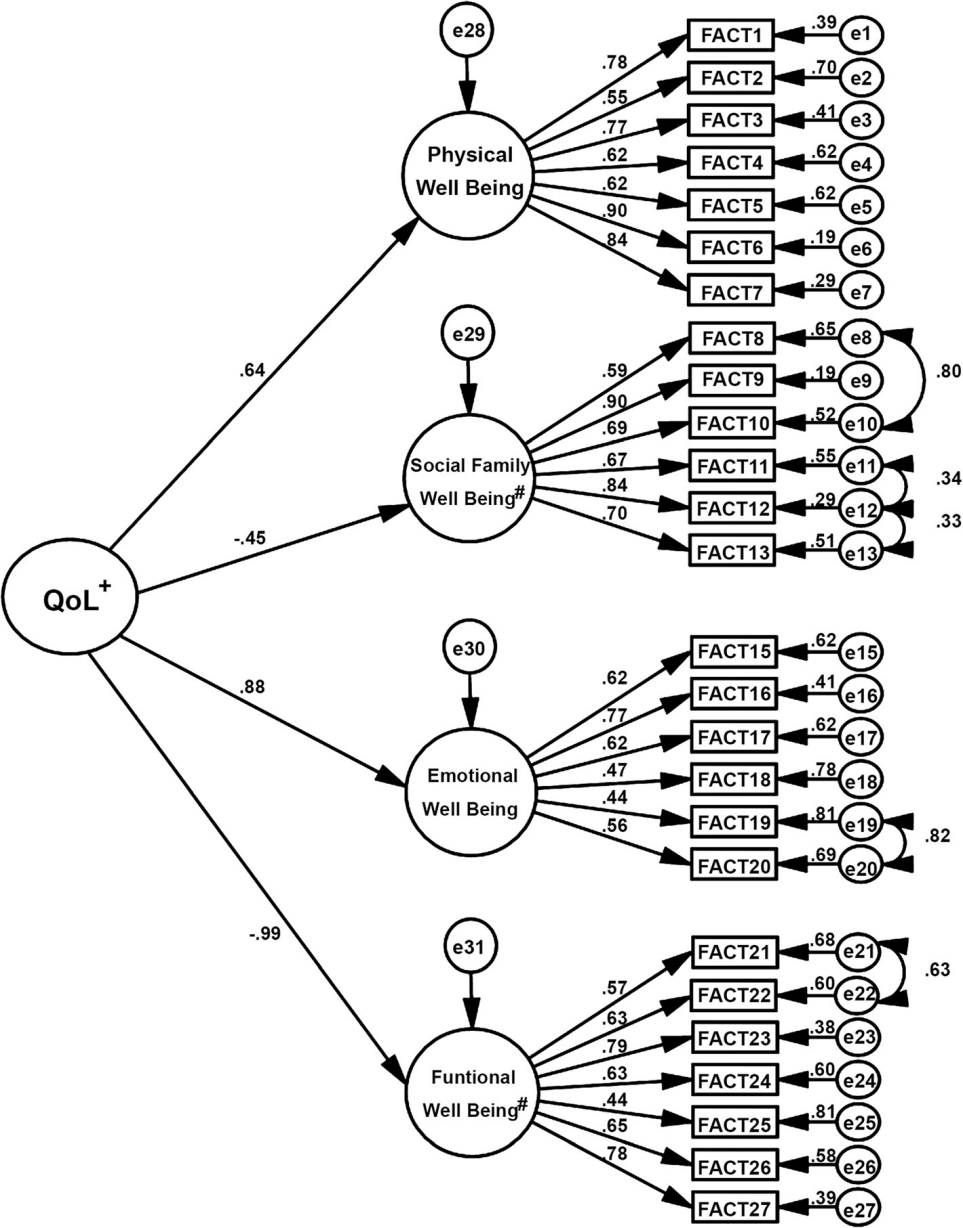
Factor structure of the second order hierarchical model of the Functional Assessment of Cancer Therapy. λ = .44 to .90; *χ* ^2^/df = 8.708, Comparative fit index (CFI) = .911, Tucker-Lewis index (TLI) = .901, Root mean square error of approximation (RMSEA) = .089 fitted to the sample of Brazilian cancer patients

The fit of the SOHM to the sample (λ = .44 to .90; *χ*^2^/df = 8.708, CFI = .911, TLI = .901, RMSEA = .089) is presented in Fig. 2.

It is noteworthy that the following factors made a great contribution to the operationalization of the HRQOL construct: Functional Well-being (β = −.985), Emotional Well-being (β = .881), and Physical Well-being (β = .641).

The correlation matrix between the FACT-G and EORTC QLQ-C30 factors is presented in Table 3. Adequate validity concurrent/divergent of FACT-G was found. Specifically, there were high correlations between theoretically similar factors, and low correlations between different factors.

**Table 3:** Correlation’s matrix of factors of the FACT-G and EORTC QLQ-C30 (version fitted to the sample)

		FACT-G				EORTC QLQ-C30									
	Factors^a^	PWB	SFWB	EWB	FWB	QL	PF	RF	EF	CF	SF	FA	NV	PA	SPUR
FACT-G	PWB	1													
	SFWB	-.17	1												
	EWB	.61	-.38	1											
	FWB	-.65	.57	-.75	1										
EORTC QLQ-C30	QL	-.61	.34	-.53	.73	1									
	PF	.87	-.14	.45	-.64	-.46	1								
	RF	.79	-.07	.44	-.70	-.49	.92	1							
	EF	.60	-.26	.90	-.51	-.47	.47	.46	1						
	CF	.49	-.21	.46	-.34	-.27	.47	.49	.42	1					
	SF	.46	-.24	.45	-.62	-.55	.47	.54	.50	.34	1				
	FA	.92	-.08	.55	-.58	-.51	.82	.81	.51	.59	.43	1			
	NV	.98	.01	.28	-.27	-.23	.43	.48	.32	.30	.21	.61	1		
	PA	1.00	-.12	0.42	-.47	-.44	.58	.61	.48	.42	.38	.68	.32	1	
	SPUR	.93	-.16	.58	-.71	-.47	.81	.78	.62	.65	.36	.98	.84	.68	1

^a^FACT-G: *PWB* physical well-being, *SFWB* social family well-being, *EWB* emotional well-being, *FWB* functional well-being; EORTC QLQ-C30: *QL* overall quality of life (global score), *PF* physical functioning, *RF* role functioning, *EF* emotional functioning, *CF* cognitive functioning, *SF* social functioning, *FA* fatigue, *NV* nausea and vomiting, *PA* pain, *SPU* spurious (single terms)

## Discussion

This study tested and determined the validity and reliability of the FACT-G when applied to a sample of Brazilian cancer patients.

The FACT-G is a widely used instrument in clinical practice for the evaluation of cancer patients. This study presented an evaluation of the instrument’s factor structure when applied to a large Brazilian sample, providing professionals with information about the steps necessary to evaluate its psychometric properties and the quality of the information that can be obtained from it.

To ensure that the structure of the FACT-G was adequate for the sample, one item was removed (item 14). In particular, item 14 pertains to the patient’s satisfaction with his/her sex life, and is therefore characterized as a very specific item that reflects the interviewee’ intimacy. As a result, the non-response rate was 45 %, which prevented its inclusion in the evaluation of the total sample. This finding was also observed by Dapueto et al. [40]. The FACIT Group suggests that if the participant does not feel comfortable responding to this item, he/she should not respond to this item and the interviewer should move to the next item. Regarding the inclusion of the correlations between the errors (LM > 11), this can be justified by the theoretical proximity of the items.

It is also clear that, although item 17 presented problems with regard to psychometric sensitivity, the same factorial structure presented adequate weight. Furthermore, the importance of its theoretical content justifies its retention for the purpose of maintaining the structure of the FACT-G. This item assesses the “hope of the individual in the fight against disease” and, as was already reported by Shinn et al. [41], is an important aspect of HRQOL.

Additionally, it is necessary to address the issue of the CVR (Table 2), as the experts only considered six FACT-G items to be essential. This raises the questions of the judges’ actual experience evaluating HRQOL, since the FACT-G was developed by a group of experts and is widely accepted at national and international levels. Thus, despite the judges’ responses in terms of items related to the construct, perhaps their knowledge is specific to particular types of cancer, leading to the determination that some instrument items were nonessential.

This study also presents a hierarchical model of the FACT-G that shows the contribution of each factor in building the central concept evaluated by the instrument. Specifically, results indicated that physical, emotional and functional well-being had a greater contribution to the construct formulation (Fig. 2). This may have been related to the high toxicity of treatments (chemotherapy and radiotherapy) and the severity of the disease, which impact patients’ physical quality of life, and consequently affect physical, functional, and emotional factors. Therefore, this should be taken into account in the approach used and/or professional management of such patients. These factors also had higher correlation coefficients in front of the EORTC QLQ-C30 including the quality of life (global) factor that aims for the same supremacy in term the impact of disease and treatment on patients’ HRQOL (Table 3).

Another aspect we found was the lack of invariance in the model when fitted to different samples. This provides a warning that, although changes in sample characteristics may not interfere with the model fit, they may interfere with the operationalization of the construct. Consequently, professionals who use this tool must know the psychometric properties of the instrument in reference to the specific sample to be evaluated.

A limitation of this study may be the adoption of a non-probabilistic sampling design, which inevitably exerted influence on sample characteristics. However, this strategy has been commonly utilized in validation studies. The use of sufficient sample size ensures credibility in the decision-making resulting from the statistical tests. Thus, it is suggested that structural model we have presented be tested in other samples, with different clinical and socio-demographic characteristics to confirm its stability and increase its representativeness. The lack of test-retest reliability could also be cited as a limitation. However, it should be considered that among patients with cancer, HRQOL may change in a short period depending on the treatment type and diagnosis. Consequently, this could skew the reproducibility of analyses. Thus, to evaluate this possibility, we suggest conducting a follow-up study. It is only by doing so that accurate evidence related to the temporal stability of the FACT-G can be obtained.

Another suggestion for future studies is to use ITR as an analytical strategy to complement traditional analyses (such as those presented in this study). The use of IRT could provide information regarding item construction, local independence, and the individual profile related to impact on HRQOL.

## Conclusion

The FACT-G was found to be a valid and reliable assessment of HRQOL in a Brazilian sample of cancer patients. It is important to note that, similar to the present study, the structure of the FACT-G should be evaluated when the sample or cultural context have distinct characteristics. This will ensure that the instrument will collect valid and reliable data suitable for each sample type.

## Abbreviations

AVE: average variance extracted
BMI: body mass index
CFA: confimatory factor analysis
CFI: comparative fit index
CR: composite reliability
CVR: content validity ratio
EORTC QLQ-C30: European Organization for Research and Treatment of Cancer-Quality of Life Questionnaire Core 30
FACIT: functional assessment of chronic illness therapy
FACT-G: functional assessment of cancer therapy-general
HRQOL: health-related quality of life
IRT: item response theory
Ku: kurtosis
RMSEA: root mean square error of approximation
LM: lagrange multipliers
MI: misunderstanding index
Sk: skewness
SOHM: second order hierarchical model
TLI: tucker-lewis index
WLSMV: weighed least squares mean and variance adjusted
WHOQOL-100: World Health Organization Quality Of Life Assessment
